# Assessing Youth Who Sexually Offended: The Predictive Validity of the ERASOR, J-SOAP-II, and YLS/CMI in a Non- Western Context

**DOI:** 10.1177/1079063211404250

**Published:** 2012-04

**Authors:** Chi Meng Chu, Kynaston Ng, June Fong, Jennifer Teoh

**Affiliations:** 1 Ministry of Community Development, Youth & Sports, Singapore; 2 Monash University, Melbourne, Australia

**Keywords:** actuarial risk prediction, criminal behavior, cross-cultural, incremental validity, recidivism, structured clinical judgment

## Abstract

Recent research suggested that the predictive validity of adult sexual offender risk assessment measures can be affected when used cross-culturally, but there is no published study on the predictive validity of risk assessment measures for youth who sexually offended in a non-Western context. This study compared the predictive validity of three youth risk assessment measures (i.e., the Estimate of Risk of Adolescent Sexual Offense Recidivism [ERASOR], the Juvenile Sex Offender Assessment Protocol-II [J-SOAP-II], and the Youth Level of Service/Case Management Inventory [YLS/CMI]) for sexual and nonviolent recidivism in a sample of 104 male youth who sexually offended within a Singaporean context (*M*_follow-up_ = 1,637 days; *SD*_follow-up_ = 491). Results showed that the ERASOR overall clinical rating and total score significantly predicted sexual recidivism but only the former significantly predicted time to sexual reoffense. All of the measures (i.e., the ERASOR overall clinical rating and total score, the J-SOAP-II total score, as well as the YLS/CMI) significantly predicted nonsexual recidivism and time to nonsexual reoffense for this sample of youth who sexually offended. Overall, the results suggest that the ERASOR appears to be suited for assessing youth who sexually offended in a non-Western context, but the J-SOAP-II and the YLS/CMI have limited utility for such a purpose.

## Introduction

Varying sexual recidivism rates have been reported in studies on youth who sexually offended (Chu & Thomas, 2010; Gretton, McBride, Hare, O’Shaughnessy, & Kumka, 2001; Långström, 2002; Nisbet, Wilson, & Smallbone, 2004; Rasmussen, 1999; Smith & Monastersky, 1996), but there is no doubt that youth sexual offending represents an ongoing issue for juvenile justice services. Mental health clinicians are often required to conduct risk assessments for youth who sexually offended, with the assumption that the clinicians can accurately predict the risk of recidivism in these youth, but this may not necessarily be the case (Caldwell, Ziemke, & Vitacco, 2008).

The meta-analytic work of Hanson and colleagues (Hanson & Bussière, 1998; Hanson & Morton-Bourgon, 2005) indicated that sexual deviance and antisociality are two domains broadly associated with sexual recidivism, but assessing the risk of sexual offending in youth may present with more challenges. It is commonly recognized that youth who offended are still developing cognitively and emotionally and that their risk status may fluctuate within a short period of time (Prentky & Righthand, 2003). In addition, more youth who sexually offended tend to recidivate nonsexually rather than sexually (Caldwell, 2007; Chu & Thomas, 2010; McCann & Lussier, 2008), suggesting that it is important to consider general antisocial antecedents in addition to sexual offending risk factors. Notwithstanding these challenges, mental health clinicians are routinely expected to provide evaluations of risk for youth who are arrested and convicted of sexual offenses. These risk assessments typically inform courts for sentencing and placement purposes and are therefore required to be accurate given the potentially serious consequences (e.g., incarceration). Equally important, the risk assessment measures play an important role in the risk management of these youth who sexually offended given the severe implications on public safety. For these purposes, structured risk assessment measures have been developed to assist clinicians with their assessments of youth who have sexually offended.

### General and Sexual Risk Assessment Measures for Assessing Youth Who Sexually Offended

In recent times, there has been more focus in the extant literature on risk assessment measures for youth who sexually offended (see Vitacco, Caldwell, Ryba, Malesky, & Kurus, 2009, for a review), but the number of studies is small when compared to the literature for risk assessment measures for their adult sexual counterparts. Given the high propensity of youth who sexually offend to engage in future nonsexual reoffenses (e.g., Caldwell, 2007; Chu & Thomas, 2010; McCann & Lussier, 2008), it is important to consider whether general risk assessment measures are accurate for assessing the risk of sexual recidivism in this group of youth, and whether sexual risk assessment measures are accurate for assessing the risk of nonsexual recidivism. One structured risk assessment measure that is commonly used for assessing the risk of general recidivism in youth who committed offenses is the Youth Level of Service/Case Management Inventory (YLS/CMI; Hoge, Andrews, & Leschied, 2002).

The YLS/CMI is primarily designed as an actuarial risk assessment measure, which is characterized by clinicians making decisions that are formulated according to explicit and fixed rules and focused on relatively small numbers of risk factors that have been determined to be predictive of recidivistic outcomes for specified settings and individuals (e.g., Quinsey, Harris, Rice, & Cormier, 2006). However, it should be noted that the YLS/CMI also allows the clinician to override the risk classification that is based on the overall score and make a final professional judgment in view of idiosyncratic and contextual factors. In terms of its predictive validity, the YLS/CMI appears to be fairly accurate for assessing general and violent recidivism in many Western contexts including Australia, Canada, the United Kingdom, and the United States (e.g., Marshall, Egan, English, & Jones, 2006; Schmidt, Hoge, & Gomes, 2005; Shepherd, Green, & Omobein, 2005; Thompson & Pope, 2005; Viljoen, Elkovitch, Scalora, & Ullman, 2009). Although Viljoen and colleagues found that the YLS/CMI did not predict sexual offending, a recent meta-analysis by Olver, Stockdale, and Wormith (2009), which included unpublished dissertations, suggested that the YLS/CMI could have some utility for such a purpose.

Pertaining to sexual risk assessment measures, the Juvenile Sex Offender Assessment Protocol-II (J-SOAP-II; Prentky & Righthand, 2003) and the Estimate of Risk of Adolescent Sexual Offense Recidivism (ERASOR; Worling & Curwen, 2001) are two widely used structured risk assessment measures for assessing risk of sexual offending in youth. Structured sexual offender risk assessment measures have been shown to be predictive of sexual recidivism for adult who have sexual offended (see Hanson & Morton-Bourgon, 2009 for a meta-analytic review), and there appears to be some support for the use of youth sexual risk assessment measures to predict both sexual and nonsexual recidivism thus far.

The J-SOAP-II is originally designed as an actuarial measure for assessing the risk of sexual offending in youth, but it is currently used as an empirically informed risk assessment guide (Prentky & Righthand, 2003). In a study of minority youth, Martinez, Flores, and Rosenfeld (2007) found that the J-SOAP-II total score significantly predict sexual recidivism (Area Under Curve [AUC] = .78) and general recidivism (AUC = .76). Similarly, the J-SOAP-II was found to significantly predict sexual and nonsexual recidivism (AUCs = .69 and .77 respectively) in a study of 286 Canadian youth who sexually offended (Rajlic & Gretton, 2010). Moreover, Prentky et al. (2010) showed that the J-SOAP-II total score was accurate for predicting sexual recidivism over 7 years for both preadolescents and adolescents who had demonstrated sexually abusive behavior, with AUCs of .80 and .83 respectively. Some studies also showed that the specific J-SOAP-II scales (e.g., Sexual Drive/Preoccupation, Impulsive/Antisocial Behavior, and Intervention), rather than the total score, predicted time to sexual reoffense (Caldwell et al., 2008; Parks & Bard, 2006; Viljoen et al., 2008). In addition, Viljoen and colleagues (2008) reported that the J-SOAP-II total score did not significantly predict sexual offending, but it predicted nonsexual aggression (AUC = .66). Furthermore, a modified scale comprising eight of nine items from the Impulsive/Antisocial Behavior scale predicted general recidivism (Waite et al., 2005). However, Elkovitch et al. (2008) found that the J-SOAP-II did not predict the sexual and nonsexual recidivism when structured clinical judgments of “low,” “moderate,” or “high” risk were used.

With regard to the ERASOR, it is a measure that is based on the structured clinical judgment approach (Worling & Curwen, 2001). This approach uses a set of empirically determined risk factors that the clinician assesses and codes; the combination of these variables and the final judgment are left to the clinician who considers the results of the risk assessment measure as well as the idiosyncratic variables that are specific to the individual being assessed (e.g., Boer, Hart, Kropp, & Webster, 1997). Worling (2004) reported in his validation study that the ERASOR total score and overall clinical ratings significantly distinguished “repeaters” (i.e., those youth who committed a sexual assault after they were caught and sanctioned by an adult) from “nonrepeaters” (i.e., those youth who did not commit further sexual assault) of sexually offensive behaviors, with AUCs of .72 and .66 respectively. More recently, Rajlic and Gretton (2010) reported similar results, with the ERASOR total score and overall clinical ratings significantly predicting sexual recidivism (AUCs = .71 and .67 respectively). Rajlic and Gretton also found that the ERASOR total score and overall clinical ratings significantly predicted nonsexual recidivism (AUCs = .71 and .70 respectively); notably, static and dynamic sections within the ERASOR predicted sexual and nonsexual recidivism. In contrast, Viljoen et al. (2009) found that the ERASOR total score did not significantly predict sexual recidivism in a sample of 193 youth who have committed offenses, and the ERASOR overall clinical rating only approached significance (AUC = .64).

### Actuarial Versus Structured Clinical Judgment Debate

There is considerable debate about predictive prowess of the actuarial and clinical judgment approaches, and the unstructured clinical judgment has been demonstrated to be less predictive than actuarial methods (see Ægisdóttir et al., 2006; Grove, Zald, Lebow, Snitz, & Nelson, 2000, for reviews). However, it has been acknowledged that structured clinical judgment measures demonstrate comparable predictive validity to actuarial tools when they are used in a mechanical actuarial manner in research (see Heilbrun, Yasuhara, & Shah, 2010, for a review). Although some studies suggest that the structured clinical judgment measures were more accurate when utilized as guides to make a structured “clinical” rating than they were used in an actuarial fashion, such conclusions must be drawn tentatively due to the small number of comparisons (Heilbrun et al., 2010). Very few studies in the youth sexual risk assessment literature have directly compared these two methods (e.g., Rajlic & Gretton, 2010; Viljoen et al., 2009) and these studies suggest that both methods have similar predictive validity for sexual recidivism.

### Cross-cultural Application of Risk Assessment Measures

Cultures and societies often define what kind of attitudes and behaviors are considered “normal” and “deviant.” Although there is some cross-cultural consensus about what constitutes sexually deviant attitudes and behaviors (e.g., molestation, rape, paraphilia, etc), definitions and development of sexually deviant attitudes and behaviors can vary significantly due to cultural norms, gender roles, mores or morals, religion, taboos, and expectations (see Bhugra, Popelyuk, & McMullen, 2010, for a review). Therefore, it is possible that risk factors for sexual offending may differ cross- culturally and will inevitably affect the utility of risk assessment measures.

In adult risk assessment literature, there were suggestions that the predictive validity of risk assessment measures (that were developed and validated in Western contexts) might be affected when they are used cross-culturally. In particular, Långström (2004) reported that the predictive validity of two adult sexual risk assessment measures (Rapid Risk Assessment of Sexual Offense Recidivism [RRASOR; Hanson, 1997], and Static-99 [Hanson & Thorton, 1999]) were different across ethnic groups in Sweden. Similarly, Allan, Dawson, and Allan (2006) found that there were differences in predictive validity for RRASOR when it is used with indigenous and nonindigenous offenders in Australia to predict sexual recidivism. Compared with the extant literature on risk assessment measures for adults who have committed sexual offenses, there are relatively few published studies on youth sexual offender risk assessment measures. Moreover, the aforementioned published studies on youth sexual offender risk assessment measures had examined only North American samples (Caldwell et al., 2008; Martinez et al., 2007; Park & Bard, 2006; Prentky et al., 2010; Rajlic & Gretton, 2010; Viljoen et al., 2008, 2009; Waite et al., 2005; Worling, 2004). To the best of our knowledge, there is currently no published study that has investigated the predictive validity of such youth risk assessment measures with a non-Western sample of youth who sexually offended.

### Youth Sexual Offenders in Singapore

Sexual offenses accounted for more than 4% of all crimes in Singapore, and these sexual offenses were mostly recorded as molestation offenses (Singapore Police Force, 2007). A recent study by Chu and Thomas (2010) reported that 11.5% of a Singaporean sample of youth who have sexually offended engaged in sexual recidivism over an average follow-up of 64.78 months. These Singaporean youth shared many similarities with youth who have sexually offended from Western countries (e.g., age of first sexual offense, criminal histories, victim characteristics, and recidivism data), but they also showed distinct differences (e.g., they are responsible for a smaller proportion of the sexual offenses, more likely to sexually assault strangers, as well as less likely to offend against male victims and be sexually victimized). Considering the cross-cultural differences in youth who sexually offended as well as suggestions that the predictive validity of these risk assessment measures may differ across cultures, the usefulness of these youth risk assessment measures in an Asian setting warrants empirical investigation.

### Present Study

There are risk management and treatment implications that are associated with risk assessments for youth who sexually offended, but there is currently limited empirical knowledge pertaining to the utility of general and sexual risk assessment measures for youth who sexually offended, especially within a non-Western context. As such, the present study sought to compare the predictive validity of the J-SOAP-II, the ERASOR, and the YLS/CMI for sexual and nonsexual recidivism in a non-Western context. In addition, the predictive validity of the actuarial and structured clinical judgment ratings was also compared. The following hypotheses were tested:

*Hypothesis 1*: Sexual offender risk assessment measures (i.e., the J-SOAP-II and the ERASOR) would have higher predictive validity for sexual recidivism than the general risk assessment measure (i.e., the YLS/CMI).
*Hypothesis 2*: The general risk assessment measure would have higher predictive validity for nonsexual sexual recidivism than the sexual offender risk assessment measures.
*Hypothesis 3*: The differences between the predictive validity of the actuarial and structured clinical judgment approaches would be nonsignificant.

## Method

### Source Sample

The sample consisted of 104 male youth who have sexually offended (aged 12 to 18 years). They were referred to the Clinical and Forensic Psychology Branch (CFPB), Ministry of Community Development, Youth and Sports (Singapore) between June 2003 and December 2007 for a psychological assessment of their risk of sexual recidivism. More than half (59.6%, 62/104) of these youth were placed on probation, whereas the remainder of the sample (40.4%, 42/104) were incarcerated in youth correctional institutions at some point during their court orders. All of the youth who sexually offended in this sample have received sexual offender treatment during the duration of their court orders.

### Procedure

The current study was a retrospective cohort follow-up study. Approval for the research study was obtained from CFPB and the Policy Branch of the Ministry of Community Development, Youth and Sports (Singapore) before the commencement of the study. For the purpose of this study, three psychologists (KN, JF, and JT) from CFPB conducted clinical file reviews, and completed the J-SOAP-II, ERASOR, and YLS/CMI ratings for the current sample of youth based on file information. The clinical files contained (a) psychological reports prepared by psychologists at CFPB, (b) presentencing reports prepared by probation officers, (c) institution risk and needs reports, (d) charge sheets, (e) statement of facts, (f) any previously existing assessment and treatment reports on the youths’ CFPB files, as well as (g) school reports. The raters were blind to the recidivism data for these youth who sexually offended. As the psychological interviews conducted at the CFPB follow a standardized semistructured interview schedule, the resultant psychological reports contain specific information pertaining to several key areas of assessment (i.e., personal, family, psychiatric, and criminal offending histories as well as the current offending behaviors and risk management issues).

The raters were trained in the application of the respective youth risk assessment measures, and had regularly conducted sexual and violent risk assessments for youth who committed criminal offenses. To examine the interrater reliability, the three raters separately coded a randomly selected sample of 16 (15.4%) files. The intraclass correlation coefficients for single rater (using absolute agreement definition; ICCs) were .77 (*excellent*) for the J-SOAP-II total score, .49 (*fair*) for the ERASOR total score, .43 (*fair*) for the ERASOR overall clinical rating, and .67 (*good*) for the YLS/CMI total score (see Cicchetti & Sparrow, 1981, for a classification of ICCs). Although the raters were trained in the usage of youth risk assessment measures, the J-SOAP-II and YLS/CMI were introduced to CFPB’s clinical practice much earlier than the ERASOR, hence higher levels of familiarity with the J-SOAP-II and YLS/CMI could have resulted to higher interrater reliability than the ERASORs.

In addition, it should be noted that the relevant risk assessment data for the ERASOR, the J-SOAP-II, and the YLS/CMI were not analyzed if there were more than two (for the ERASOR and the J-SOAP-II) and five (for the YLS/CMI) omitted items respectively; only two cases had more than two omitted items for the J-SOAP-II. Furthermore, recidivism data were obtained from the Criminal Records Office, Singapore Police Force (on April 6, 2010) on the completion of the initial risk assessment ratings and file review, and were coded by a research assistant. Recidivism was coded in the following way with a record made as to whether each youth engaged in sexual recidivism (e.g., indecent exposure, molestation, peeping, rape, and sodomy) and/or nonsexual recidivism (i.e., any offenses that were not classified as sexual in nature) during the follow-up.

### Youth Risk Assessment Measures

#### ERASOR

The ERASOR is an empirically guided, structured clinical judgment measure that comprises 25 items (16 dynamic and 9 static risk factors) and is designed to assist clinicians in estimating the risk of sexual recidivism for youth (aged 12 to 18 years) who have presented with sexual offending behaviors (Worling & Curwen, 2001). The items are grouped into five sections: Sexual Interests, Attitudes, and Behaviors; Historical Sexual Assaults; Psychosocial Functioning; Family/Environmental Functioning; and Treatment. Each item can be coded as Unknown, Not Present, Possibly/Partially Present, or Present, which were assigned numerical values of 0, 1, 2, and 3 respectively for purpose of actuarial comparisons. Clinically, the ERASOR does not apply cutoff scores or formulas for determining the risk level, rather the evaluators make an overall clinical rating (i.e., structured professional rating/judgment) of Low, Moderate, or High risk. However, in this study, we have examined both the overall clinical ratings and the total score that was derived from summing the item scores.

#### J-SOAP-II

The J-SOAP-II is a 28-item structured assessment checklist that is used to review the risk factors that have been empirically associated with sexual and criminal offending in youth (Prentky & Righthand, 2003). It is intended for use with male youth who are aged between 12 and 18 years, and has four scales: Sexual Drive/Preoccupation, Impulsive and Antisocial Behavior, Intervention, and Community Stability/Adjustment. All the 28 items are coded on a 3-point scale where 0 indicates *an absence of the risk factor*, 1 suggests *some evidence of the factor being present*, and 2 indicates *a clear presence of the factor or greater degree*. The scores can be summed to obtain the total score.

#### YLS/CMI

The YLS/CMI is a structured assessment instrument designed to facilitate the effective intervention and rehabilitation of youth who have committed criminal offenses (aged 12 to 18 years) by assessing their risk level and criminogenic needs (Hoge et al., 2002). It consists of 42 items (scored as either Present or Absent) that are divided into eight subscales (Prior or Current Offenses/Dispositions, Family Circumstances/Parenting, Education/Employment, Peer Relations, Substance Abuse, Leisure/Recreation, Personality/Behavior, and Attitudes/Orientation). The item scores (i.e., the number of indicated risk factors/needs) can be aggregated to obtain a total risk/needs score. The YLS/CMI was used strictly as an actuarial measure in the present study, thus the risk classification and professional override features of the YLS/CMI were not examined.

### Statistical Analyses

The sample was characterized using descriptive statistics, with categorical data reported as numbers and percentages, and continuous data presented in relation to the mean and standard deviation. In addition, Pearson *r* correlations were used to examine the correlations between the scores on the risk assessment measures. Receiver Operating Characteristic (ROC) analyses were also undertaken to examine the predictive validity of the risk assessment measures during the follow-up. The ROC, which generates an AUC, is a commonly used method for examining the predictive validity of risk assessment measures, and it is less dependent on the base rates of reoffending than traditional measures of predictive accuracy (Douglas & Webster, 1999). To compare the AUCs of ROC Curves for total scores and overall clinical rating, *z* tests for dependent groups (Hanley & McNeil, 1983) were used to ascertain whether the AUCs differed significantly between the risk assessment measures for each follow-up period. Critical ratio z is defined as: $Z=(A_{1}-A_{2})=\sqrt{SE_{1}^{2}+SE_{2}^{2}-2rSE_{1}SE_{2}}$ and *z* values of ≥ |1.96| were taken as evidence that the “true” areas under the ROC Curves were different. Benjamini and Hochberg False Discovery Rate (FDR) corrections were also conducted to control for Type I error that may arise from multiple comparisons; specifically, it is a less conservative but more powerful statistical approach than Bonferroni-type adjustments (Benjamini & Hochberg, 1995).

In addition, Cox regression models were developed to examine whether the risk assessment measures significantly predicted time to reoffense after accounting for the length of incarceration. Furthermore, when two or more measures were found to significantly predict a certain type of recidivism, stepwise logistic regression analysis was conducted to evaluate the incremental validity. Specifically, risk measure A was entered in Step 1, which was followed by risk measure B in Step 2. This order was then reversed to investigate whether risk measure A and/or risk measure B explained any unique variance (e.g., Viljoen et al., 2009; Welsh, Schmidt, McKinnon, Chatta, & Meyers, 2008), and changes in Nagelkerke *R*^2^ were used to examine incremental validity. Analyses were carried out using the PASW Statistics version 18.

## Results

### Sample Characteristics

The mean age of these youth at referral to CFPB and at first reported sexual offense were 15.16 years (*SD* = 1.44; range = 12 to 18) and 14.28 years (*SD* = 1.58; range = 9 to 18) respectively. The mean length of the follow-up was 1,637 days (*SD* = 491), but varied from 817 to 2,741 days. The large majority of the youth who sexually offended were Chinese (41.3%, 43/104) or Malay (44.2%; 46/104); 12.5% (13/104) were Indian, and 2% (2/104) were of other ethnicity. Three quarters had committed molestation (76%, 79/104), 9.6% (10/104) committed rape, and 19.2% (20/104) committed other sexual offenses (e.g., nonconsensual fellatio, peeping, and indecent exposure). Eight of the 104 (7.7%) youth reoffended sexually, and 27 (26.0%) recidivated nonsexually.

### Total Scores of the Risk Assessment Measures

The mean total scores of the ERASOR, the J-SOAP-II, and the YLS/CMI were 36.84 (*SD* = 6.21; range = 24 to 52), 20.27 (*SD* = 7.90; range = 5 to 39), and 14.23 (*SD* = 7.22; range = 0 to 29) respectively. In addition, 28.8% (30/104) were rated as Low risk on the ERASOR (overall clinical rating), 50% (52/104) as Moderate risk, and 21.2% (22/104) as High risk.

### Correlations Between the Risk Assessment Measures

Table 1 summarizes the correlations between the ERASOR, the J-SOAP-II, and the YLS/CMI. The total scores (and the overall clinical rating) of all the risk assessment measures were significantly correlated with one another, and these correlations were moderate to large (see Cohen, 1988).

**Table 1.** Correlations Between Risk Assessment Measures

	ERASOR		J-SOAP-II	YLS/CMI
Measure	Total score	OCR	Total score	Total score
ERASOR (*N* = 104)				
Total score	—	.82***	.70***	.48***
Overall clinical rating (OCR)	.82***	—	.68***	.35***
J-SOAP-II total score (*N* = 102)	.70***	.68***	—	.71***
YLS/CMI total score (*N* = 104)	.48***	.35***	.71***	—

*** *p* < .001.

### Predictive Validity of the Risk Assessment Measures and Subscales/Sections

#### Predictive validity of measures

Table 2 presents the predictive accuracy of risk assessment measures for sexual and nonsexual recidivism during the follow-up period. ROC analyses revealed that the ERASOR overall clinical rating (AUC = .83, *SE* = .07, 95% confidence interval [95% CI] = [.70, .96], *p* = .002) and the ERASOR total score (AUC = .74, *SE* = .07, 95% CI = [.61, .88], *p* = .02) significantly predicted sexual recidivism during the follow-up period even after FDR corrections. Of note, the J-SOAP-II total score (AUC = .51, *SE* = .09, 95% CI = [.33, .69], *ns*) and the YLS/CMI total score (AUC = .29, *SE* = .08, 95% CI = [.15, .44], *ns*) did not significantly predict sexual recidivism. Further comparisons revealed that the ERASOR overall clinical rating and total score were significantly better than the J-SOAP-II and the YLS/CMI at predicting sexual recidivism (*z*_ERASOR_OCR-JSOAP_ = |3.71|, *p* < .001; *z*_ERASOR_OCR-YLS_ = |5.90|, *p* < .001; *z*_ERASOR_Tot-JSOA_ = |2.76|, *p* = .006; *z*_ERASOR_Tot-YLS_ = |5.27|, *p* < .001). Moreover, the ERASOR overall clinical rating was significantly better than the ERASOR total score at predicting sexual recidivism (*z* = |2.34|, *p* = .019).

**Table 2.** The Predictive Validity of ERASOR, J-SOAP-II and YLS/CMI for Sexual and Nonsexual Recidivism

	Sexual recidivism		Nonsexual recidivism	
Risk assessment measure	AUC (*SE*)	95% CI	AUC (*SE*)	95% CI
ERASOR (*N* = 104)				
Total score	.74* (.07)^a^	[.61, .88]	.66* (.06)^a^	[.54, .78]
Overall clinical rating	.83**(.07)^a^	[.70, .96]	.69**(.06)^a^	[.58, .80]
Sexual interests, attitudes, & behaviors	.64 (.10)	[.45, .83]	.67* (.06)	[.54, .79]
Historical sexual assaults	.81** (.06)^a^	[.70, .92]	.54 (.06)	[.42, .67]
Psychosocial functioning	.49 (.08)	[.33, .65]	.58 (.07)	[.44, .71]
Family/environmental functioning	.49 (.11)	[.28, .70]	.66* (.06)^a^	[.54, .77]
Treatment	.55 (.11)	[.33, .78]	.51 (.07)	[.38, .63]
J-SOAP-II (*N* = 102)				
Total score	.51 (.09)	[.33, .69]	.79*** (.06)^a^	[.68, .89]
Sexual drive/ preoccupation	.72* (.08)^a^	[.56, .89]	.52 (.07)	[.39, .66]
Impulsive/antisocial behavior	.37 (.10)	[.18, .56]	.71** (.06)^a^	[.60, .82]
Intervention	.41 (.10)	[.22, .60]	.79***(.05)^a^	[.68, .89]
Community stability/ adjustment	.55 (.11)	[.34, 76]	.69** (.06)^a^	[.58, .81]
YLS/CMI (*N* = 104)				
Total score	.29 (.08)	[.15, .44]	.65* (.06)^a^	[.53, .76]
Offenses/disposition	.43 (.10)	[.24, .61]	.51 (.07)	[.38, .64]
Family circumstances & parenting	.37 (.12)	[.13, .60]	.54 (.06)	[.42, .66]
Education/employment	.31 (.08)	[.15, .47]	.66* (.06)	[.54, .79]
Peer relations	.31 (.10)	[.12, .50]	.62 (.06)	[.50, .73]
Substance abuse	.50 (.11)	[.29, .71]	.48 (.06)	[.36, .61]
Lesisure/recreation	.56 (.10)	[.35, .76]	.57 (.06)	[.44, .69]
Personality/behavior	.24 (.08)	[.09, .40]	.63* (.06)	[.52, .75]
Attitudes/orientation	.36 (.10)	[.16, .55]	.66* (.06)	[.54, .79]

a. Denotes that the risk assessment measure significantly predicted recidivistic outcome even after Benjamini and Hochberg False Discovery Rate (FDR) corrections.

* *p* < .05. ** *p* < .01 ***p < .001.

Notwithstanding the FDR corrections, all of the risk assessment measures significantly predicted nonsexual recidivism during the follow-up (AUC_JSOAP_ = .80, *SE* = .07, 95% CI = [.66, .94], *p* = .001; AUC_YLS/CMI_ = .65; *SE* = .06, 95% CI =[ .53, .76], *p* = .025; AUC_ERASOR_OCR_ = .69, *SE* = .06, 95% CI = [.58, .80], *p* = .003; AUC_ERASOR_Tot_ = .66, *SE* = .06, 95% CI = [.54, .78], *p* = .012). In addition, the J-SOAP-II was significantly better than the other measures at predicting nonsexual recidivism (*z*_JSOAP-ERASOR_OCR_ = |1.99|, *p* = .047; *z*_JSOAP-ERASOR_Tot_ = |2.84|, *p* = .005; *z*_JSOAP-YLS_ = |2.75|, *p* = .006), but the differences between the other measures were nonsignificant.

#### Predictive validity of measures’ subscales/sections

Table 2 shows the predictive validity of the measures’ subscales for sexual and nonsexual recidivism. Only the Historical Sexual Assaults section from the ERASOR (AUC = .81, *SE* = .06, 95% CI = [.70, .92], *p* = .004), and the Sexual Drive/Preoccupation scale from the J-SOAP-II (AUC = .72, *SE* = .08, 95% CI = [.56, .89], *p* = .038) significantly predicted sexual recidivism after FDR corrections; whereas Impulsive/Antisocial Behavior (AUC = .71, *SE* = .06, 95% CI = [.60, .82], *p* = .001), Intervention (AUC = .79, *SE* = .05, 95% CI = [.68, .89], *p* < .001), and Community Stability/Adjustment (AUC = .69, *SE* = .06, 95% CI = [.58, .81], *p* = .003) scales from the J-SOAP-II, as well as the Family/Environmental Functioning section from the ERASOR (AUC = .66, *SE* = .06, 95% CI = [.54, .77], *p* = .014) significantly predicted nonsexual recidivism after FDR corrections.

#### Predicting time to reoffense

Cox regression analyses revealed that the ERASOR overall clinical rating (*B* = 2.34, *SE* = .77, Wald = 9.32, *p* = .002, Exp[*B*] = 10.43, 95% CI = [2.31, 46.96]) significantly predicted time to sexual reoffense after accounting for time at risk, but the ERASOR total score (*B* = 0.12, *SE* = .06, Wald = 4.27, *p* = .039, Exp[*B*] = 1.12, 95% CI = [1.01, 1.26]) did not (following FDR correction). In addition, the J-SOAP-II total score (*B* = 0.02, *SE* = .05, Wald = 0.13, *ns*, Exp[*B*] = 1.02, 95% CI = [.93, 1.11]) and the YLS/CMI total score (*B* = -0.09, *SE* = .06, Wald = 2.54, *ns*, Exp[*B*] = 0.92, 95% CI = [.82, 1.02]) also did not predict time to sexual reoffense. However, all of the risk assessment measures significantly predicted time to nonsexual reoffense (the ERASOR overall clinical rating: B = 1.00, *SE* = .29, Wald = 11.93, *p* = .001, Exp[*B*] = 2.71, 95% CI = [1.54, 4.76]; the ERASOR total score: *B* = 0.08, *SE* = .03, Wald = 6.75, *p* = .009, Exp[*B*] = 1.08, 95% CI = [1.02, 1.15]; the J-SOAP-II total score: *B* = 0.12, *SE* = .02, Wald = 24.07, *p* < .001, Exp[*B*] = 1.13, 95% CI = [1.07, 1.18]; the YLS/CMI total score: *B* = 0.08, *SE* = .03, Wald = 7.87, *p* = .005, Exp[*B*] = 1.08, 95% CI = [1.02, 1.14]).

#### Incremental validity of measures

As the ERASOR overall clinical rating and total score were the only measures that significantly predicted sexual recidivism, no further analyses were undertaken to examine incremental validity with the other measures. Instead, analyses were undertaken to determine whether the ERASOR overall clinical rating and ERASOR total score contributed any unique variance when used together. The ERASOR overall clinical rating (*B* = 4.24, *SE* = 1.47, Wald = 8.30, *p* = .004, Exp[*B*] = 69.38, 95% CI = [3.87, 1,242.34], Δ*R*^2^ = .27) was the only significant predictor when it was entered into model with the ERASOR total score (*B* = -0.23, *SE* = .42, Wald = 2.66, *ns*, Exp[*B*] = 0.79, 95% CI = [0.60, 1.05], Δ*R*^2^ = .06).

All of the measures showed significant predictive validity for nonsexual recidivism, therefore they were entered into logistic regression models to examine their incremental validity. Tables 3, 4, and 5 show the incremental validity for the risk assessment measures for nonsexual recidivism. Notably, the J-SOAP-II total score remained the only significant predictor even after the other measures were entered into the regression equation subsequently, and it also yielded the most incremental validity when it was entered into Steps 2 and 3 of the logistic regression models.

**Table 3:** Incremental Validity of Measures for Nonsexual Recidivism (With J-SOAP-II as 1st Predictor)

		*B*	*SE*	Wald	*p*	Exp(*B*)	95% CI	Δ*R*^2^
Step 1								
J-SOAP-II		0.14	.04	14.20	< .001	1.15	[1.07, 1.23]	—
Step 2								
J-SOAP-II^1^ – ERASOR_ OCR^2^	(1)	0.11	.04	6.60	.010	1.12	[1.03, 1.22]	
	(2)	0.42	.48	0.76	.380	1.52	[0.59, 3.89]	<.01
J-SOAP-II^1^ – ERASOR_ Total^2^	(1)	0.17	.05	10.49	.001	1.18	[1.07, 1.31]	
	(2)	−0.06	.06	0.82	.367	0.95	[0.84, 1.07]	.01
J-SOAP-II^1^ – YLS/CMI^2^	(1)	0.16	.05	10.89	.001	1.17	[1.07, 1.29]	
	(2)	−0.04	.05	0.54	.462	0.97	[0.88, 1.06]	<.01
Step 3								
J-SOAP-II^1^ – ERASOR_ OCR^2^ – YLS/ CMI^3^	(1)	0.13	.06	5.17	.023	1.14	[1.02, 1.28]	
	(2)	0.35	.50	.50	.480	1.42	[0.54, 3.74]	
	(3)	−0.03	.50	.30	.587	0.97	[0.88, 1.07]	<.01
J-SOAP-II^1^ – ERASOR_ Total^2^ – YLS/ CMI^3^	(1)	0.19	.06	9.80	.002	1.21	[1.07, 1.36]	
	(2)	−0.06	.06	0.81	.369	0.95	[0.84, 1.07]	
	(3)	−0.04	.05	0.53	.465	0.96	[0.87, 1.06]	<.01
J-SOAP-II^1^ – YLS/CMI^2^ – ERASOR_ OCR^3^	(1)	0.13	.06	5.17	.023	1.14	[1.02, 1.28]	
	(2)	−0.03	.05	0.30	.587	0.97	[0.88, 1.07]	
	(3)	0.35	.50	0.50	.480	1.42	[0.54, 3.74]	<.01
J-SOAP-II^1^ – YLS/CMI^2^ – ERASOR_ Total^3^	(1)	0.19	.06	9.80	.002	1.21	[1.07, 1.36]	
	(2)	−0.04	.05	0.53	.465	0.96	[0.87, 1.06]	
	(3)	−0.06	.06	0.81	.369	0.95	[0.84, 1.07]	.01

*Note*. ^1^ = First predictor, ^2^ = Second predictor, ^3^ = Third predictor

**Table 4.** Incremental Validity of Measures for Nonsexual Recidivism (With ERASOR as 1st Predictor)

		*B*	*SE*	Wald	*p*	Exp(*B*)	95% CI	Δ*R*^2^
ERASOR_OCR entered as 1st predictor								
Step 1								
ERASOR_OCR		1.15	.36	9.96	.002	3.16	[1.55, 6.45]	—
Step 2								
ERASOR_OCR^1^ – J-SOAP-II^2^	(1)	0.42	.48	0.76	.384	1.52	[0.59, 3.89]	
	(2)	0.11	.04	6.60	.010	1.12	[1.03, 1.22]	.09
ERASOR_OCR^1^ – YLS/CMI^2^	(1)	1.03	.38	7.32	.007	2.81	[1.33, 5.92]	
	(2)	0.05	.04	2.07	.151	1.05	[0.98, 1.13]	.03
Step 3								
ERASOR_OCR^1^ – J-SOAP-II^2^ – YLS/CMI^3^	(1)	0.35	.50	0.50	.480	1.42	[0.54, 3.74]	
	(2)	0.13	.06	5.17	.023	1.14	[1.02, 1.28]	
	(3)	−0.03	.05	0.30	.587	0.97	[0.88, 1.07]	<.01
ERASOR_OCR^1^ – YLS/CMI^2^ – J-SOAP- II^3^	(1)	0.35	.50	0.50	.480	1.42	[0.54, 3.74]	
	(2)	−0.03	.05	0.30	.587	0.97	[0.88, 1.07]	
	(3)	0.13	.06	5.17	.023	1.14	[1.02, 1.28]	.07
ERASOR_Total entered as 1st predictor								
Step 1								
ERASOR_Total		0.09	.04	5.04	.025	1.09	[1.01, 1.17]	—
Step 2								
ERASOR_Total^1^ – J-SOAP-II^2^	(1)	−0.06	.06	0.82	.367	0.95	[0.84, 1.07]	
	(2)	0.17	.05	10.49	.001	1.18	[1.07, 1.31]	.17
ERASOR_Total^1^ – YLS/CMI^2^	(1)	0.06	.04	1.88	.171	1.06	[0.98, 1.15]	
	(2)	0.05	.04	2.06	.152	1.05	[0.98, 1.13]	.03
Step 3								
ERASOR_Total^1^ – J-SOAP-II^2^ – YLS/CMI^3^	(1)	−0.06	.06	0.81	.369	0.95	[0.84, 1.07]	
	(2)	0.19	.06	9.80	.002	1.21	[1.07, 1.36]	
	(3)	−0.04	.05	0.53	.465	0.96	[0.87, 1.06]	<.01
ERASOR_Total^1^ – YLS/CMI^2^ – J-SOAP- II^3^	(1)	−0.06	.06	0.81	.369	0.95	[0.84, 1.07]	
	(2)	−0.04	.05	0.53	.465	0.96	[0.87, 1.06]	
	(3)	0.19	.06	9.80	.002	1.21	[1.07, 1.36]	.15

*Note*. ^1^ = First predictor, ^2^ = Second predictor, ^3^ = Third predictor

**Table 5:** Incremental Validity of Measures for Nonsexual Recidivism (With YLS/CMI as 1st Predictor)

		*B*	*SE*	Wald	*p*	Exp(*B*)	95% CI	Δ*R*^2^
Step 1								
YLS/CMI		0.08	.03	5.21	.022	1.08	[1.01, 1.15]	—
Step 2								
YLS/CMI^1^ – J-SOAP-II^2^	(1)	−0.04	.05	0.54	.462	0.97	[0.88, 1.06]	
	(2)	0.16	.05	10.89	.001	1.17	[1.07, 1.29]	.17
YLS/CMI^1^ – ERASOR_OCR^2^	(1)	0.05	.04	2.07	.151	1.05	[0.98, 1.13]	
	(2)	1.03	.38	7.32	.007	2.81	[1.33, 5.92]	.10
YLS/CMI^1^ – ERASOR_Total^2^	(1)	0.05	.04	2.06	.152	1.05	[0.98, 1.13]	
	(2)	0.06	.04	1.88	.171	1.06	[0.98, 1.15]	.03
Step 3								
YLS/CMI^1^ – J-SOAP-II^2^ – ERASOR_OCR^3^	(1)	−0.03	.05	0.30	.587	0.97	[0.88, 1.07]	
	(2)	0.13	.06	5.17	.023	1.14	[1.02, 1.28]	
	(3)	0.35	.50	0.50	.480	1.42	[0.54, 3.74]	<.01
YLS/CMI^1^ – J-SOAP-II^2^ – ERASOR_Total^3^	(1)	−0.04	.05	0.53	.465	0.96	[0.87, 1.06]	
	(2)	0.19	.06	9.80	.002	1.21	[1.07, 1.36]	
	(3)	−0.06	.06	0.81	.369	0.95	[0.84, 1.07]	.01
YLS/CMI^1^ – ERASOR_OCR^2^ – J-SOAP- II^3^	(1)	−0.03	.05	0.30	.587	097	[0.88, 1.07]	
	(2)	0.35	.50	0.50	.480	1.42	[0.54, 3.74]	
	(3)	0.13	.06	5.17	.023	1.14	[1.02, 1.28]	.07
YLS/CMI^1^ – ERASOR_Total^2^ – J-SOAP- II^3^	(1)	−0.04	.05	0.53	.465	0.96	[0.87, 1.06]	
	(2)	−0.06	.06	0.81	.369	0.95	[0.84, 1.07]	
	(3)	0.19	.06	9.80	.002	1.14	[1.07, 1.36]	.15

*Note*. ^1^ = First predictor, ^2^ = Second predictor, ^3^ = Third predictor

## Discussion

The present study examined the predictive validity of the ERASOR, the J-SOAP-II, and the YLS/CMI for sexual and nonsexual recidivism in a sample of 104 youth who sexually offended within a Singaporean context. In addition, the study also examined the predictive validity of the actuarial and structured clinical judgment risk assessment approaches.

### Predicting Sexual Recidivism

Of the risk assessment measures that were examined, the ERASOR demonstrated the most promise in predicting sexual recidivism in the Singaporean context. In particular, only the ERASOR overall clinical rating significantly predicted sexual recidivism and time to recidivism during the course of follow-up. Given that the average follow-up in this study was 4.5 years, this finding seems to somewhat contradict Worling’s (2004) suggestion that “the final risk estimate derived from the ERASOR is short-term (i.e., at most 1 year) and should not be used to address questions related to long-term risk” (p. 239), especially since the ERASOR is comprised predominantly of dynamic items and thus arguably more suited for shorter term predictions of recidivism (see Chu, Thomas, Ogloff, & Daffern, 2010, for a comparison of static and dynamic risk assessment measures)

A possible explanation could be that a group of static risk factors on the ERASOR (i.e., Historical Sexual Assaults section), rather than the dynamic sections, contributed significantly to the prediction of sexual recidivism over the 4.5-year follow-up. In particular, it would be interesting to examine the predictive validity of these dynamic risk factors over shorter time periods in future studies. Although dynamic risk factors may continue to operate as risk factors over a long period of time, the fluctuating nature of these factors may affect the predictive validity of these risk factors for recidivistic outcomes over longer term (especially using single time-point assessment methodology; see Douglas & Skeem, 2005 for a review).

The present study showed that the ERASOR’s predictive validity for sexual recidivism (using overall clinical rating and total score) was somewhat higher than those reported by Rajlic and Gretton (2010), Viljoen et al. (2009), and Worling (2004) with youth of similar ages in Western contexts, but it is clear that the ERASOR has acceptable to excellent predictive utility (see Hosmer & Lemeshow, 2000 for a classification of AUCs) for sexual recidivism beyond the shorter-term for youth who sexually offended from Western and non-Western contexts.

In contrast, the J-SOAP-II’s predictive validity for sexual recidivism was poor; specifically, its total score did not significantly predict sexual recidivism and the time to sexual reoffense. Closer examination of the J-SOAP-II scales revealed that only the Sexual Drive/Preoccupation scale showed significant predictive validity for sexual recidivism following FDR corrections. Apart from this finding on the Sexual Drive/Preoccupation scale, the present findings on the J-SOAP-II were generally inconsistent with the extant literature, which found that the J-SOAP-II total score and several subscales had significantly predicted sexual recidivism (Caldwell et al., 2008; Martinez et al., 2007; Prentky et al., 2010; Rajlic & Gretton, 2010; Viljoen et al., 2008). Consistent with the findings from Viljoen et al. (2009), neither the YLS/CMI total score nor its subscales significantly predict sexual recidivism in this study. Taken together, there is partial support for the first hypothesis; in particular, the ERASOR (a sexual risk assessment measure) has demonstrated better predictive validity for assessing youth sexual offenders in a non-Western context than the YLS/CMI (a general risk assessment measure), but the J-SOAP-II (a sexual risk assessment measure) did not.

### Predicting Nonsexual Recidivism

With regard to the predictive validity for nonsexual recidivism, the J-SOAP-II showed the most promise, but the other risk assessment measures were also rather accurate at predicting nonsexual recidivism (see Table 2). These findings provided support for Viljoen et al.’s (2008) findings regarding the J-SOAP-II (i.e., it was more suited for predicting nonsexual recidivism rather than sexual recidivism). Similar to Viljoen et al.’s (2009) findings, the YLS/CMI was found to be suited for predicting nonsexual recidivism in this study. It was further noted that, consistent with Rajlic and Gretton’s (2010) findings, Impulsive/Antisocial Behavior, Intervention, and Community Stability/Adjustment scales of the J-SOAP-II significantly predicted nonsexual recidivism, suggesting that these scales comprised of factors that strongly associated with general antisocial orientation. However, the current findings did not support Viljoen et al.’s (2009) findings regarding the ERASOR’s poor predictive validity for nonsexual recidivism. Instead, the present study showed that the ERASOR was fairly accurate for predicting nonsexual recidivism and time to nonsexual reoffense. It was further noted that the J-SOAP-II was significantly more predictive of nonviolent recidivism than a general risk assessment measure such as the YLS/CMI.

In terms of incremental validity, the ERASOR overall clinical rating and J-SOAP-II total score added unique variance when used with the YLS/CMI to predict nonsexual recidivism. Of note, the YLS/CMI did not significantly predict nonsexual recidivism when used together with the J-SOAP-II and the ERASOR—a finding that questions the suitability of using general risk assessment measure when assessing a youth who sexually offended. More importantly, the J-SOAP-II accounted for most of the variance when it is used together with other measures, suggesting that it is most suited for assessing the risk of nonsexual recidivism in youth who sexually offended. Considering that youth who sexually offend are more likely to recidivate nonsexually than sexually (e.g., Caldwell, 2007; Chu & Thomas, 2010; McCann & Lussier, 2008), it is perhaps unsurprising that existing youth sexual risk assessment measures aim to measure both sexual and nonsexual violence, and they may even perform adequately when predicting nonsexual recidivism (as found in this study and others; e.g., Viljoen et al., 2008, 2009). Overall, the results from this study do not provide support for the second hypothesis.

### Actuarial Versus Structured Clinical Judgment Ratings

In contrast to Rajlic and Gretton (2010) as well as Viljoen et al. (2009), our findings suggest that structured clinical judgment method (i.e., the ERASOR overall clinical rating) was more accurate than the actuarial methods (the ERASOR, YLS/CMI, and J-SOAP-II total scores) when predicting sexual recidivism. Notably, the ERASOR overall clinical rating also showed significant incremental validity when used with the ERASOR total score. However, the picture is less clear when we examined the predictive validity of these approaches for nonsexual recidivism. Specifically, the predictive validity of the J-SOAP-II total score for nonsexual recidivism was significantly better than the ERASOR overall clinical rating, which was expected since the latter pertained specifically to the risk of sexual reoffending. Nevertheless, the differences between the ERASOR overall clinical rating and the other actuarial measures (i.e., the ERASOR and YLS/CMI total scores) were nonsignificant. Taken together, there is mixed support for the third hypothesis.

One possible explanation for this observation is that the raters had incorporated or weighed information (specific to sexual offending) differently in this sample than might have occurred in a Western sample. For example, if the raters are aware of culturally relevant behaviors, attitudes, or risk factors that are not given adequate weight in the actuarial process, they can compensate for this shortcoming when using a structured clinical judgment measure. Hence, the present findings may also reflect a subtle form of cultural bias in the data that leads to less accurate actuarial predictions for sexual recidivism within the Singapore context.

### Cross-cultural Application of the Risk Assessment Measures

There were suggestions that in adult sexual offender literature that the sexual risk assessment measures were not equally accurate across ethnic groups even within the same Western context (Allan et al., 2006; Långström, 2004). In addition, although there were similarities in characteristics between the youth who sexually offended in Singapore and their Western counterparts, there were also distinct differences (Chu & Thomas, 2010). Collectively, these studies raised a question as to whether sexual risk assessment measures that were developed in Western contexts are applicable in non-Western contexts (e.g., Singapore). For example, it is possible that some of the risk factors (e.g., sexually assaulting strangers) that are included in the youth sexual risk assessment measures (e.g., the J-SOAP-II and the ERASOR) may not relate directly to those present in youth who sexually offended within the Singaporean context and thus may not discriminate the sexual recidivists from the nonrecidivists as effectively. The consideration of less relevant risk factors may ultimately affect the predictive validity of the risk assessment measures. Furthermore, culture-specific risk factors need to be considered, and it will be advantageous to operationalize the coding criteria of the risk factors from the various measures according to the contexts in which the measures will be used.

From a clinical perspective, the findings from this study show that the ERASOR is generally suited for predicting sexual and nonsexual recidivism in a Singaporean context. Although the J-SOAP-II and the YLS/CMI showed significant predictive validity for nonsexual recidivism, clinicians should exercise caution when using the J-SOAP-II to assess the risk of sexual recidivism in youth within the Singaporean context (in particular, only the Sexual Drive/Preoccupation scale showed significant predictive validity for sexual recidivism) and avoid using the YLS/CMI for such a purpose. Notwithstanding that the YLS/CMI have limited use for assessing youth who sexually offended, its Case Management Inventory may offer a good platform for risk management and intervention planning whereby assessment findings from clinical interviews and other risk assessment measures can be incorporated. In addition to risk prediction/classification, another major goal of risk assessment is risk reduction (Heilbrun, 1997). Therefore, it may be beneficial to consider the following when developing risk management and rehabilitation plans for youth who sexually offended in a non-Western context: (a) whether the same risk factors, criminogenic needs, and responsivity factors (see Andrews & Bonta, 2007, for a review on these factors) operate in various cross-cultural contexts; (b) whether these can be accurately determined by the existing risk assessment measures; and (c) the additional culture-specific risk, needs, and responsivity factors.

### Limitations and Future Research

First, the sample size of the present study is relatively small and, similar to most studies on youth who sexually offended (see Caldwell, 2010, for a review), the base rate for sexual recidivism obtained from official records was rather low. Although we relied on the electronic data and archival file data for recidivism follow-up, there would inevitably be an underestimate of offending due to the further offenses not having been detected. Furthermore, the predictive validity of these assessment measures relative to nonsexual offending was based on a sample of youth initially convicted of sexual offenses; thus these results may not generalize to youth who had committed only nonsexual offenses initially. Notwithstanding these limitations, it should be noted that this study had employed statistical analyses (e.g., ROC analyses) that are less dependent on base rates of reoffending and, coupled with appropriate analyses (e.g., FDR corrections) that can address Type I error without being overly conservative, we can therefore be relatively confident about the results. Moreover, this study has yielded much needed information on the predictive validity and applicability of such risk assessment measures for youth who sexually offended in a non-Western context, and has provided a springboard for further comparative studies in such a context.

Future research on youth sexual risk assessment measures should employ prospective and repeated measures designs in which the risk assessments are based on interviews as well as information that is available in archival records. Moreover, it is beneficial to examine the validity of such measures for predicting sexual and nonsexual recidivism in female youth who sexually offended, as there is currently very little information on the applicability of these risk assessment measures for this population.
